# Mutations in hepatitis B virus polymerase are associated with the postoperative survival of hepatocellular carcinoma patients

**DOI:** 10.1371/journal.pone.0189730

**Published:** 2017-12-29

**Authors:** Fei Yin, Ying Xie, Haiyan Fan, Jingjing Zhang, Zhanjun Guo

**Affiliations:** 1 Department of Gastroenterology and Hepatology, The Fourth Hospital of Hebei Medical University, Shijiazhuang, P.R. China; 2 Hebei Key Lab of Laboratory Animal Science, Hebei Medical University, Shijiazhuang, P.R. China; National Health Research Institutes, TAIWAN

## Abstract

Proofreading deficiencies of hepatitis B virus polymerase result in frequent DNA mutations in the hepatitis B virus genome. Here, we performed sequencing analysis of the hepatitis B virus polymerase gene to assess its association with the postoperative survival in 92 patients with HBV-related hepatocellular carcinoma by using the Kaplan–Meier method. The 2525, 2733, 2738, 2768, 2946, 3063, 3066, 3109, 31, 529, 735, 939, 1078, 1137, 1383, 1461, 1485, 1544, and 1613 mutation sites were identified as being associated with HCC outcomes by the log-rank test. After adjusting for clinical characteristics by using the Cox hazard model, site 31 (relative risk, 8.929; 95% confidence interval, 3.433–23.22; *P* = 0.000) in the spacer domain and sites 529 (relative risk, 5.656; 95% confidence interval, 1.599–19.999; *P* = 0.007) and 1078 (relative risk, 3.442; 95% confidence interval, 1.070–11.068; *P* = 0.038) in the reverse transcriptase domain of hepatitis B virus polymerase were identified as independent predictors of postoperative survival in hepatitis B virus related hepatocellular carcinoma. The mutations at the 31 (Ser314Pro), 529 (Asp480Asn), and 1078 (Ser663Ala) sites all resulted in amino acid changes in hepatitis B virus polymerase and were associated with shortened life-span. The 31 and 529 sites were located in the overlapping region for the PreS and S genes but did not induce amino acid substitution in these two regions. Our finding of the correlation between hepatitis B virus DNA polymerase mutations and hepatocellular carcinoma survival will help identify the patients subgroup with poor prognosis, and help the clinicians to refine the therapeutic decision individualized.

## Introduction

Hepatocellular carcinoma (HCC) is the sixth most common malignancy and the third leading cause of cancer-related deaths worldwide[1,2]. Hepatitis B virus (HBV) infection, hepatitis C infection, alcohol consumption, and aflatoxins are the main risk factors for HCC. HBV infection accounts for 80% of HCC cases, especially in developing countries[3,4]. In China, more than 93 million people are HBV carriers and about 30 million have chronic hepatitis B[5]. Although the currently available diagnostic and treatment methods have improved greatly, the postoperative prognosis of patients with HCC is still poor because of the high recurrence rate. Some of the associated clinical features such as tumor size, tumor quantity, cell differentiation, venous invasion, and viral characteristics have been identified as predictors of and prognostic factors for HCC, but the true genetic mechanism underlying this disease remains unknown[6–11].

The HBV genome encodes four proteins with an overlapping open reading frame: surface protein (S), core protein (C), polymerase, and a multifunctional nonstructural protein called X (HBx). HBV polymerase is the only enzymatically active protein; it participates in many steps of the viral replication cycle, such as encapsidation, packaging, nucleocapsid assembly (minus strand DNA synthesis), and pregenomic RNA degradation[12,13]. HBV polymerase can be divided into four sections: a terminal protein (TP) domain, a reverse transcriptase (RT) domain, an RNase H-like (RH) domain, and a spacer domain[14]. Among them, the RT and RH domains are necessary for polymerase function. A previous study indicated that the A799G, A987G, and T1055A mutations in the RT domain are predictive markers of HCC occurrence[15].

Proofreading deficiencies of HBV polymerase result in HBV DNA hypermutation[16,17]. In addition to HBV genotype and viral DNA load, mutations in the preS1, preS2, basic core promoter, and enhacer II (EnhII) regions have also been found to be associated with HBV-related HCC (HBV-HCC) risk and HCC development[18–23]. We have previously shown the prognostic value of some mutations in the HBx region and PreC/C region for patients with HBV-HCC[24,25]. In this study, we assessed the prognostic value of mutations in the polymerase gene for postoperative survival; for this purpose, we sequenced the HBV polymerase region by using tumor tissue specimens from patients with HBV-HCC. And finally the results indicated that three mutations were linked to the postoperative survival of HBV-HCC patients.

## Materials and methods

### Tissue specimens

96 resected tumor tissue specimens were collected from patients who had been diagnosed with HCC and had undergone surgical resection between 2007 and 2009 at the Department of Hepatobiliary Surgery, Fourth Hospital of Hebei University, China. Patients with HCV or autoimmune hepatitis were excluded. All procedures were supervised and approved by the Human Tissue Research Committee of the Fourth Hospital of Hebei University and were in accordance with the principles expressed in the Declaration of Helsinki. Informed written consent was obtained from each patient.

### DNA extraction, real-time polymerase chain reaction (PCR), and sequencing

Genomic DNA was extracted using the DNA FFPE Tissue Kit (Qiagen, Hilden, Germany) as per the manufacturer’s instructions. And the extracted DNA was stored at -80°C until it was analyzed. The HBV DNA concentration was quantified as copies per microgram of genomic DNA by real-time PCR performed using the ABI 7300 TaqMan platform (Life Technologies, Carlsbad, CA, USA); multiplex PCR was used to determine the HBV genotype[26]. The DNA sequences flanking the polymerase region (nucleotides 2307–3215, 1–1623) were amplified with the primer pairs listed in Table 1, according to the polymerase region identified by the National Center for Biotechnology Information (NCBI) database (http://www.ncbi.nlm.nih.gov/genome/5536). It was very difficult to obtain PCR products for some samples; therefore, we redesigned the primer pairs with decreased flanking length for successful PCR amplification in such cases. Nested PCR was also performed for a few samples.

**Table 1:** Primer pairs used to amplify and sequence the HBV polymerase regions.

Primer	Nucleotide sequence (5’→3’)	Position	
**HBV-P-1F**	5' CCCGCTTACAGACCACCAAA 3'	**2288–2307**	**Sense**
**HBV-P-1R**	5' AAATTGAGAGAAGTCCACCACGAGT 3'	**252–277**	**Anti-sense**
**HBV-P-2F**	5' CGTGTTACAGGCGGGGTTTT 3'	**193–213**	**Sense**
**HBV-P-2R**	5' AGGTCGGTCGTTGACATTGCT 3'	**1676–1696**	**Antisense**

Cyclic sequencing was performed with a BigDye Terminator v3.1 Cycle Sequencing Kit (Life Technologies), and the products were separated using an ABI PRISM 3100 Genetic Analyzer (Life Technologies). Mutations were confirmed by repeating the analysis on both strands.

### Statistical analysis

Kaplan–Meier survival curves were generated, and comparisons between these curves were made using the log-rank test. Multivariate survival analysis was performed using a Cox proportional hazards model. All the statistical analyses were performed using the SPSS 18.0 software package (IBM Corporation, Armonk, NY, USA), and a *P*-value of <0.05 was considered statistically significant.

## Results

### Clinical characteristics of patients with HBV-HCC

A total of 96 patients with HBV-HCC were enrolled in this study and were followed up every 3 months for 3 years. The HBV genotype of these patients with HBV-HCC was assessed and was as follows: one patient, genotype A; one patient, genotype D; two patients, genotype B+C; 52 patients, genotype B; and 40 patients, genotype C. The data for patients with the HBV B or C genotype were subsequently used for survival analysis with the Kaplan–Meier method. Portal vein thrombosis (*P* = 0.000), tumor size (*P* = 0.002), tumor-node-metastasis (TNM) classification (*P* = 0.001), and HBV DNA load (*P* = 0.026) were found to be associated with the postoperative survival of patients with HBV-HCC by using the log-rank test (Table 2). After performing adjustments with the Cox proportional hazards model, the multivariate analysis indicated that portal vein thrombosis was an independent predictor of survival of patients with HBV-HCC (RR, 2.728; 95% confidence interval [CI], 1.322–5.631; *P* = 0.007).

**Table 2:** Univariate and multivariate analysis of the clinical factors associated with postoperative survival in HBV-HCC patients.

Factors	No. of	3-year	Univariate analysis	Multivariate analysis		
	case	survival rate	*P*-value	*P-*value	RR^a^	95%CI
**Age**			**0.664**	**0.744**	**1.104**	**0.609–2.001**
**≤55**	**50**	**48.0%**				
**>55**	**42**	**45.7%**				
**Gender**			**0.719**	**0.564**	**1.307**	**0.527–3.239**
**Female**	**11**	**45.5%**				
**Male**	**81**	**45.7%**				
**CHILD** **classification**			**0.123**	**0.241**	**1.743**	**0.689–4.408**
**A**	**84**	**47.6%**				
**B**	**8**	**25.0%**				
**Genotype**			**0.425**	**0.745**	**1.100**	**0.619–1.955**
**B**	**52**	**50.0%**				
**C**	**40**	**40.0%**				
**Tumor quantity**			**0.126**	**0.101**	**1.884**	**0.883–4.019**
**Single**	**77**	**48.1%**				
**Multiple**	**15**	**33.3%**				
**Portal vein thrombosis**			**0.000**	**0.007**	**2.728**	**1.322–5.631**
**Yes**	**13**	**7.7%**				
**No**	**79**	**51.9%**				
**TNM classification**			**0.001**	**0.810**	**1.138**	**0.397–3.263**
**I**	**34**	**67.6%**				
** II+ III**	**58**	**32.8%**				
**Tumor size (diameter)**			**0.002**	**0.167**	**1.857**	**0.772–4.464**
**≤5cm**	**40**	**62.5%**				
**>5cm**	**52**	**32.7%**				
**HBV DNA**^**b**^			**0.026**	**0.247**	**1.490**	**0.759–2.926**
**≧1×10**^**7**^	**66**	**53.0%**				
**< 1×10**^**7**^	**26**	**26.9%**				

^a^RR: Relative Risk

^b^Defined as copies per microgram of genomic DNA

### Virological parameters associated with the postoperative survival of patients with HCC

**Fig 1:**
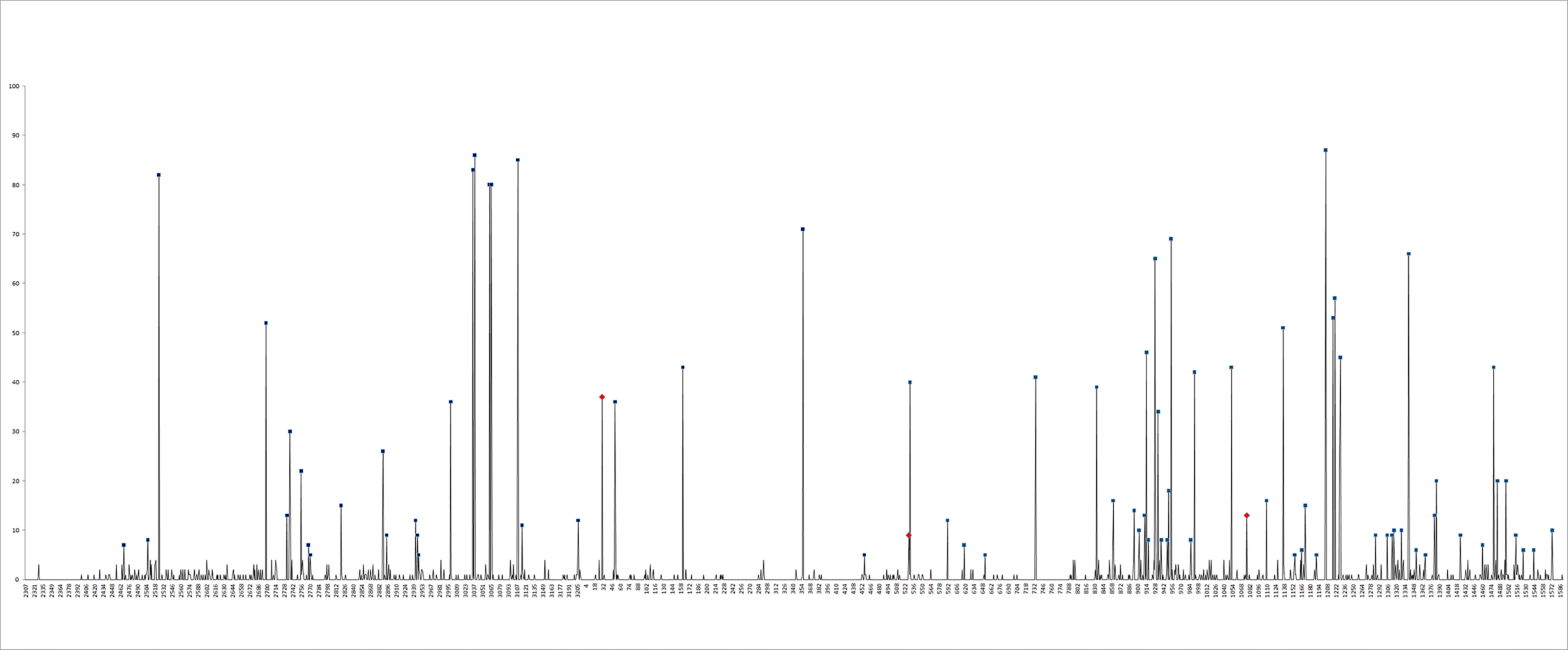
Distribution of mutation frequency in the HBV polymerase region. The square indicates the mutations associated with survival in HBV-HCC, as determined by univariate analysis using the log-rank test; the rhombus indicates mutations that are not associated with HBV-HCC.

HBV DNA was extracted from the HCC tissue for virological analysis; the mean HBV DNA level was 4.66 × 10^9^ ± 1.60 × 10^10^ copies/μg, with a maximum copy number of 1.33 × 10^11^ and a minimum copy number of 1.25 × 10^5^. The DNA sequences flanking the polymerase region were sequenced; in patients with HCC, a total of 86 mutation sites with mutation rates >5% were used for survival analysis (Fig 1). The highest mutation rate was observed at the 1206 nucleotide position in 87 patients who carried this mutation (Fig 1).

**Fig 2:**
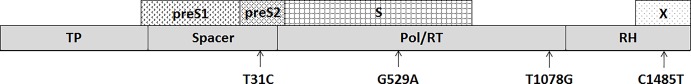
Schema of the polymerase region and overlapping preS, S, and X regions.

Of these 86 sites, the following 19 were associated with postoperative survival at statistically significant levels (P<0.05) in HCC tissue based on the log-rank test with the Kaplan–Meier method (Table 3): nucleotides 2525 (Asp73Glu), 2733 (Lys143Gln), 2738, 2768, 2946 (Val210Ile), 3063, 3066 (Pro249Ser), 3109 (Thr268Ser), 31 (Ser314Pro), 529 (Asp480Asn), 735, 939, 1078 (Ser663Ala), 1137, 1383, 1461, 1485, 1544 (Val818Asp or Val818Ala); and 1613 (Arg841Lys). These sites were located in the following domains of HBV polymerase: the 2525, 2733, 2738 and 2768 sites were located in the TP domain; 2946, 3063, 3066, 3109, and 31 in the spacer domain; 529, 735, 939, and 1078 in the RT domain; and 1137, 1383, 1461, 1485, 1544, and 1613 in the RH domain. Almost a quarter of the detected mutations (19/86) were associated with overall survival in HCC, and only three of them (3/19) were associated with worse outcomes by multivariate analysis (Fig 1 and Table 4) based on the Cox proportional hazards model in HBV-HCC patients. As shown in Table 4, after adjusting for clinical characteristics, the following three mutation sites were identified as independent predictors of a shorter survival period in HCC at statistically significant levels: mutation at nucleotide 31 (RR, 8.929; 95% CI, 3.433–23.22; *P* = 0.000) inducing the 314 Ser to Pro amino acid substitution in the spacer domain; at nucleotide 529 (RR, 5.656; 95% CI, 1.599–19.999; *P* = 0.007) inducing the 480 Asp to Asn amino acid substitution; and at nucleotide 1078 (RR, 3.442; 95% CI, 1.070–11.068; *P* = 0.038) inducing the 663 Ser to Ala amino acid substitution in the RT domain of HBV polymerase (Table 3). The nucleotide 31 and 529 mutation sites were located in the region of HBV polymerase overlapping with preS/S; they did not induce amino acid substitution in the PreS and S regions (Fig 2).

**Table 3:** Univariate analysis of HBV polymerase gene mutations associated with postoperative survival in patients with HBV-HCC.

Nucleotide	No. of	3-year	Univariate	Amino acid	Nucleotide	No. of	3-year	Univariate	Amino acid
Site	case	survival	analysis	substitution	Site	case	survival	analysis	substitution
		rate(%)					rate(%)		
**2468**			**0.782**		**929**			**0.613**	
**t**	**85**	**45.9**			**a**	**27**	**51.9**		
**c**	**7**	**42.9**			**t**	**65**	**43.1**		
**2507**			**0.925**		**930**			**0.806**	
**a**	**84**	**45.2**			**a**	**77**	**33.3**		
**other**	**8**	**50.0**			**t or g**	**15**	**46.5**		
**2525**			**0.017**		**934**			**0.903**	
**t**	**10**	**20.0**		**Asp-Glu**	**c**	**58**	**46.6**		
**g**	**82**	**48.8**		**D73E**	**a**	**34**	**44.1**		
**2699**			**0.153**		**939**			**0.000**	
**g**	**40**	**37.5**			**g**	**84**	**50.0**		**No**
**other**	**52**	**51.9**			**a**	**8**	**0.0**		
**2708**			**0.897**		**949**			**0.802**	
**t**	**88**	**45.5**			**c**	**84**	**45.2**		
**other**	**4**	**50.0**			**a**	**8**	**50.0**		
**2733**			**0.001**		**951**			**0.734**	
**a**	**79**	**50.6**		**Lys-Gln**	**a**	**74**	**44.6**		
**c**	**13**	**15.4**		**K143Q**	**g**	**18**	**50.0**		
**2737**			**0.772**		**955**			**0.931**	
**c**	**79**	**45.6**			**t**	**23**	**43.5**		
**t**	**13**	**46.2**			**c**	**69**	**46.4**		
**2738**			**0.004**		**987**			**0.387**	
**t**	**62**	**35.5**		**No**	**a**	**84**	**44.0**		
**c**	**30**	**66.7**			**c**	**8**	**62.5**		
**2756**			**0.197**		**993**			**0.257**	
**t**	**70**	**41.4**			**a**	**50**	**40.0**		
**c**	**22**	**59.1**			**g**	**42**	**52.4**		
**2768**			**0.021**		**1053**			**0.781**	
**t**	**85**	**48.2**		**No**	**g**	**49**	**46.9**		
**g**	**7**	**14.3**			**a**	**43**	**44.2**		
**2771**			**0.649**		**1078**			**0.000**	
**a**	**87**	**46.0**			**t**	**79**	**50.6**		**Ser-Ala**
**g**	**5**	**40.0**			**g**	**13**	**15.4**		**S663A**
**2821**			**0.372**		**1110**			**0.876**	
**c**	**77**	**42.9**			**c**	**76**	**44.7**		
**a**	**15**	**60.0**			**t**	**16**	**50.0**		
**2889**			**0.708**		**1137**			**0.004**	
**g**	**66**	**43.9**			**a**	**41**	**61.0**		
**a**	**26**	**50.0**			**g**	**51**	**33.3**		
**2895**			**0.187**		**1156**			**0.783**	
**t**	**83**	**43.4**			**c**	**87**	**46.0**		
**g**	**9**	**66.7**			**a**	**5**	**40.0**		
**2943**			**0.096**		**1167**			**0.506**	
**t**	**80**	**42.5**			**a**	**86**	**46.5**		
**g**	**12**	**66.7**			**c**	**6**	**33.3**		
**2946**			**0.002**		**1173**			**0.625**	
**g**	**83**	**49.4**		**Val-Ile**	**c**	**77**	**46.8**		
**a**	**9**	**11.1**		**V210I**	**t**	**15**	**40.0**		
**2948**			**0.155**		**1191**			**0.783**	
**t**	**87**	**43.7**			**c**	**87**	**46.0**		
**c**	**5**	**80.0**			**t**	**5**	**40.0**		
**3000**			**0.236**		**1206**			**0.616**	
**c**	**56**	**50.0**			**t**	**5**	**40.0**		
**t or a**	**36**	**38.9**			**a**	**87**	**46.0**		
**3036**			**0.639**		**1218**			**0.955**	
**t**	**9**	**55.6**			**c**	**39**	**46.2**		
**c**	**83**	**44.6**			**t**	**53**	**45.3**		
**3039**			**0.402**		**1221**			**0.278**	
**t**	**6**	**33.3**			**a**	**35**	**40.0**		
**g**	**86**	**46.5**			**t**	**57**	**49.1**		
**3063**			**0.019**		**1229**			**0.211**	
**a**	**12**	**25.0**		**No**	**g**	**66**	**42.4**		
**c**	**80**	**48.8**			**a**	**26**	**53.8**		
**3066**			**0.008**		**1230**			**0.830**	
**c**	**12**	**25.0**		**Pro-Ser**	**g**	**47**	**46.8**		
**t**	**80**	**48.8**		**P249S**	**c**	**45**	**44.4**		
**3109**			**0.000**		**1287**			**0.203**	
**c**	**7**	**0.0**		**Thr-Ser**		**83**	**43.4**		
**g**	**85**	**49.4**		**T268S**		**9**	**66.7**		
**3207**			**0.186**		**1306**			**0.309**	
**g**	**80**	**48.8**			**c**	**83**	**47.0**		
**a**	**12**	**25.0**			**a**	**9**	**33.3**		
**31**			**0.000**		**1314**			**0.283**	
**t**	**55**	**69.1**		**Ser-Pro**	**a**	**83**	**43.4**		
**c**	**37**	**10.8**		**S314P**	**g**	**9**	**66.7**		
**52**			**0.157**		**1317**			**0.963**	
**t**	**56**	**51.0**			**g**	**82**	**45.1**		
**c**	**36**	**36.1**			**a**	**10**	**50.0**		
**162**			**0.858**		**1329**			**0.534**	
**g**	**49**	**44.9**			**a**	**82**	**46.3**		
**a**	**43**	**46.5**			**c**	**10**	**40.0**		
**357**			**0.473**		**1341**			**0.069**	
**c**	**21**	**52.4**				**26**	**30.8**		
**t**	**71**	**43.7**				**66**	**51.5**		
**457**					**1353**			**0.727**	
**a**	**87**	**44.8**	**0.407**			**86**	**45.3**		
**g**	**5**	**60.0**				**6**	**50.0**		
**529**			**0.003**		**1368**			**0.186**	
**g**	**86**	**48.8**		**Asp-Asn**		**87**	**43.7**		
**a**	**6**	**0.0**		**D480N**		**5**	**80.0**		
**531**			**0.077**		**1383**			**0.025**	
**t**	**52**	**40.4**			**a**	**79**	**40.5**		**No**
**c**	**40**	**52.5**			**c**	**13**	**76.9**		
**592**			**0.171**		**1386**			**0.357**	
**c**	**80**	**42.5**			**c**	**22**	**36.4**		
**t**	**12**	**66.7**			**g**	**70**	**48.6**		
**619**			**0.596**		**1425**			**0.586**	
**c**	**85**	**45.9**			**c**	**83**	**44.6**		
**t**	**7**	**42.9**			**t**	**9**	**55.6**		
**653**			**0.498**		**1461**			**0.016**	
**t**	**87**	**44.8**			**g**	**85**	**48.2**		**No**
**a**	**5**	**60.0**			**others**	**7**	**14.3**		
**735**			**0.027**		**1479**			**0.639**	
**c**	**51**	**52.9**		**No**	**c**	**29**	**41.4**		
**t**	**41**	**36.6**			**a or g**	**43**	**47.6**		
**834**			**0.974**		**1485**			**0.012**	
**c**	**53**	**47.2**			**c**	**72**	**51.4**		**No**
**a**	**39**	**43.6**			**t**	**20**	**25.0**		
**861**			**0.056**		**1499**			**0.432**	
**c**	**76**	**48.7**			**a**	**72**	**47.2**		
**t**	**16**	**31.3**			**t or g**	**20**	**40.0**		
**895**			**0.979**		**1515**			**0.442**	
**t**	**78**	**46.2**			**g**	**83**	**44.6**		
**g**	**14**	**42.9**			**a**	**9**	**55.6**		
**903**			**0.917**		**1527**			**0.099**	
**t**	**82**	**46.3**			**c**	**86**	**43.0**		
**a**	**10**	**40.0**			**t**	**6**	**83.3**		
**912**			**0.943**		**1544**			**0.015**	
**g**	**79**	**45.6**			**t**	**86**	**47.7**		**Val-Asp or Ala**
**a**	**13**	**46.2**			**a or c**	**6**	**16.7**		**V818D or A**
**915**			**0.946**		**1574**			**0.312**	
**t**	**46**	**45.7**			**a**	**82**	**43.9**		
**c**	**46**	**45.7**			**t**	**10**	**60.0**		
**918**			**0.151**		**1613**			**0.000**	
**a**	**84**	**42.9**			**g**	**74**	**54.1**		**Arg-Lys**
**g**	**8**	**75.0**			**a**	**18**	**11.1**		**R841K**

**Table 4:** Multivariate analysis of the clinical factors associated with postoperative survival in HBV-HCC patients.

Factors	Multivariate analysis		
	*P*-value	RR^a^	95%CI
Age	0.718	1.158	0.523–2.564
Gender	0.974	1.021	0.287–3.633
CHILD classification	0.042	4.445	1.053–18.768
Genotype	0.996	0.997	0.398–2.500
Tumor quantity	0.064	2.649	0.944–7.439
Portal vein thrombosis	0.028	3.321	1.137–9.699
TNM classification	0.313	1.554	0.659–3.663
Tumor size (diameter)	0.525	0.717	0.258–1.997
HBV DNA^b^	0.363	1.607	0.578–4.472
2525	0.579	0.712	0.214–2.370
2733	0.168	2.672	0.661–10.808
2738	0.626	0.776	0.279–2.156
2768	0.531	0.668	0.189–2.358
2946	0.160	2.781	0.669–11.565
3063	0.533	1.873	0.260–13.487
3066	0.122	0.239	0.039–1.469
3109	0.599	1.995	0.152–26.187
31	0.000	8.929	3.433–23.22
529	0.007	5.656	1.599–19.999
735	0.951	1.035	0.352–3.039
939	0.132	2.828	0.732–10.928
1078	0.038	3.442	1.070–11.068
1137	0.680	1.229	0.461–3.273
1383	0.269	0.388	0.072–2.078
1461	0.111	3.201	0.765–13.399
1485	0.061	2.461	0.960–6.309
1544	0.808	1.226	0.237–6.345
1613	0.971	1.020	0.360–2.886

^a^RR: Relative Risk

We evaluated the potential correlation between these three mutations and clinical characteristics, including gender, age, TNM stage, tumor size, tumor number, portal vein thrombosis, HBV levels, HBV genotype, serum alpha-fetoprotein levels, and serum hepatobiliary enzyme level by the chi-square test. The nucleotide 31 mutation site was found to be associated with alpha-fetoprotein levels (*P* = 0.018) and Tumor size (*P* = 0.005), whereas the 529 site was associated with TNM stage (*P* = 0.029) (S1 Table). Furthermore, one mutation site was associated with survival at a borderline significance level: 1485 (RR, 2.461; 95% CI, 0.960–6.309; *P* = 0.061), without amino acid substitution in the RH domain (Table 3).

## Discussion

HBV infection remains a major health challenge in developing countries as it is highly prevalent[27]. Virological factors such as HBV DNA load, genotype, and nucleotide mutations have been identified to be linked with hepatocarcinogenesis[28]. Some mutations in the HBx, basic core promoter, EnHII, and Pre C/C regions were found to be predictive factors for hepatocarcinogenesis, but only a few actually hold prognostic value in patients with HBV-HCC[14]. We previously identified HBV mutations 1383, 1461, 1485, 1544, 1613, 1653, 1719, and 1753 in the HBx and 1915, 2134, 2221, 2245 and 2288 in PreC/C regions were associated with the survival of patients with HBV-HCC[24,25]. In the current study, mutations in the HBV polymerase region were evaluated, and multivariate analysis showed that three mutations were associated with survival in HBV-HCC. Our results showed that amino acid substitution mutations at the 31, 529, and 1078 nucleotides were linked to the postoperative prognosis of patients with HCC, while the association of nucleotide 1485 was at borderline levels of statistical significance. To our knowledge, this study is the first to show that HBV polymerase mutations are associated with the postoperative survival times of patients with HCC.

G529A and T1078G were located in the RT domain of polymerase, which possesses RT and DNA-dependent DNA polymerase activity[14]. The lamivudine resistance–linked G529A (rtD134N) site in HBV was found to be associated with HCC outcomes, which implied potential correlation between resistance to the anti-HBV nucleoside analog lamivudine and HCC prognosis[29]. The G529A mutation did not result in a structural change in HBsAg, which could induce changes in the antigenic properties of HBV[30,31]. T1078G, which is located in the non-overlapping region of the RT domain, can induce an amino acid change from Ser to Ala (rtS316A). Further functional research is required to determine whether the amino acid residue changes induced by G529A and T1078G would result in altered RT activity so as to modify HBV replication and cell proliferation in HCC. T31C, located in the spacer domain, is a highly variable region of polymerase. The spacer domain was identified as having no detrimental effects on replication competence. C1485T, which is located in the RH domain overlapping with HBx, possesses RNase H activity that can degrade the pregenomic RNA template during minus-strand DNA elongation[32]. Our previous study identified C1485T as a prognostic factor, which causes a critical amino acid change (Pro38Ser) in the X protein[25]. To our knowledge, this is the first study to report that the mutations identified in the spacer and RH domains have prognostic value in HCC.

In conclusion, a total of four mutations in the HBV polymerase gene were identified as predictors of the postoperative survival of patients with HCC. Further studies need to be conducted in laboratory settings to determine the mechanisms underlying this relationship, while verifying the results in a larger population. Here, we regard these HBV DNA mutations as being independently predictive of the postoperative survival of patients with HCC. Analysis of HBV DNA mutations may help identify patient subgroups with poor prognosis, and the findings may help the clinicians to refine the therapeutic decision individualized.

## Supporting information

S1 Table Association of the HBV polymerase mutations and serum biological features. (XLS)
